# Development of Novel Lightweight Metastable Metal–(Metal + Ceramic) Composites Using a New Powder Metallurgy Approach

**DOI:** 10.3390/ma13153283

**Published:** 2020-07-23

**Authors:** Khin Sandar Tun, Akshay Padnuru Sripathy, Sravya Tekumalla, Manoj Gupta

**Affiliations:** 1 Department of Mechanical Engineering, National University of Singapore, Singapore 119077, Singapore; mpekhst@nus.edu.sg (K.S.T.); akshayps@u.nus.edu (A.P.S.); 2 School of Mechanical and Aerospace Engineering, Nanyang Technological University, Singapore 639798, Singapore; sravya.tekumalla@ntu.edu.sg

**Keywords:** magnesium, powder metallurgy, microstructure, mechanical properties

## Abstract

In the current study, metal–(metal + ceramic) composites composed of biocompatible elements, magnesium (Mg), zinc (Zn), calcium (Ca) and manganese (Mn) were synthesized using a sinter-less powder metallurgy method. The composite has a composition of Mg_49_Zn_49_Ca_1_Mn_1_ (wt.%) in which the compositional ratio between Mg and Zn was chosen to be near eutectic Mg-Zn composition. The synthesis method was designed to avoid/minimize intermetallic formation by using processing temperatures lower than the Mg-Zn binary eutectic temperature (~ 340 °C). The synthesis process involved extrusion of green compacts at two different temperatures, 150 °C and 200 °C, without sintering. Extrusion was performed directly on the green compacts as well as on the compacts soaked at temperatures of 150 °C and 200 °C, respectively. Microstructure and mechanical properties of the materials synthesized under various processing conditions were investigated. Effect of extrusion temperature as well as soaking temperature on the materials’ properties were also evaluated in details and different properties showed an optimum under different conditions. All the synthesized materials showed no evidence of intermetallic formation which was confirmed by SEM/EDS, XRD, and Differential Scanning Calorimetry (DSC) techniques. The study establishes development of unconventional metal–(metal + ceramic) eco-friendly composites and provides important insight into realizing certain properties without using sintering step thus to minimize the energy consumption of the process. The study also highlights the use of magnesium turnings (recyclability) to develop advanced materials.

## 1. Introduction

Magnesium is well known as the lightest structural metal. Mg-based materials are gaining continual interest due to their usefulness in a wide range of weight critical applications such as in automotive, aerospace, electronics, maritime, defense, and sports sectors. In addition, Mg is one of the most biocompatible metals, naturally present in bone, and is also one of the essential elements for metabolism [1]. Mg based alloys and composites are considered as promising biodegradable materials for orthopedic and cardiovascular applications [2]. Compared to other metallic biomaterials, Mg possesses similar mechanical properties to human bones, excellent biocompatibility, and lower density compared to currently used biomaterials such as titanium and steels [2,3]. However, pure magnesium has posed challenges to be used in biomedical applications due to its high corrosion in high chloride environment of physiological system [4]. Hence, alloying, composite technology, and surface coatings were used to enhance biomedical performance of magnesium [5,6,7]. Alloying technique is often selected as an effective way to improve the mechanical properties and corrosion resistance of Mg. Recently, researchers focused on the investigation of biologically safe Mg alloys, comprising of non-toxic elements such as Ca, Zr, Zn, and Mn [8,9]. Zinc, one of the bioabsorbable metals and the most essential nutrient elements in the human body, is a popular alloying element in Mg [2]. The increased addition of Zn in Mg led to enhancement of tensile strengths up to 6 wt.% of Zn addition [10]. However, the addition of Zn beyond 4 wt.% showed the formation of second phases which causes the deterioration of corrosion resistance in Mg-Zn alloys. Yan et al. [11] also reported the presence of second phases in Mg-Zn alloys with Zn content of 6.3 wt.% and corrosion resistance accelerated with immersion time. Ca and Mn are mostly added to Mg-Zn alloys to mitigate the corrosion resistance of Mg-Zn alloy without causing strength decrement [12,13,14,15]. Similar to Zn being an excellent choice of alloying element to Mg, Mg is also a promising alloying element to Zn [16]. From the previous investigations [17,18,19], Zn-Mg alloys exhibit good corrosion resistance, enabling them to be promising candidates for implant applications.

For materials processing, two main processing routes—liquid metallurgy and powder metallurgy—are used. In case of liquid metallurgy such as casting, it is difficult to control the formation of intermetallic phases in metal alloy fabrication due to high processing temperature well above the melting temperatures of constituent alloying elements. In case of powder metallurgy, intermetallic formation can be controlled or minimized as the processing temperature is usually below melting temperature. Especially for the fabrication of biomaterials, it is important to have a control over contamination and intermetallic formation which are two critical sources for degradation and materials’ biomedical performances such as biocorrosion [10,11].

In the current investigation, metal–(metal + ceramic) composites were synthesized using sinter-less powder metallurgy (PM) method with a process flow of cold compaction followed by hot extrusion with and without soaking (homogenizing heat treatment) prior to extrusion. The sinter-less PM approach was aimed to develop metal–(metal + ceramic) eco-friendly composites without formation of intermetallic compounds. The composition of the metal–(metal + ceramic) composites was chosen as Mg_49_Zn_49_Ca_1_Mn_1_ (wt.%). The composition of Zn (49 wt.%) was selected close to the eutectic Mg-Zn composition (51.3 wt.%). This enables easy identification of the eutectic phase (Mg_7_Zn_3_) [20]. Equal composition for Mg and Zn, 49 wt.% each, was also used to enable the impetus for the development of either Mg-based or Zn-based biocompatible materials using current PM approach. In the newly designed processing method, the extrusion temperatures (150 °C and 200 °C) lower than the Mg-Zn binary eutectic temperature (~ 340 °C) were used in order to avoid/minimize the formation of intermetallic compounds. The soaking temperatures prior to extrusion as well as extrusion temperatures were selected to be 150 °C which is the onset of magnesium’s recrystallization temperature and 200 °C which is 50 °C above recrystallization temperature, respectively. The effects of processing parameters (soaking temperature and extrusion temperature) and processing conditions (soaking and non-soaking prior to extrusion) on microstructure evolution, hardness, tensile, and compressive properties were investigated.

## 2. Materials and Methods

### 2.1. Materials

The Mg_49_Zn_49_Mn_1_Ca_1_ (wt.%) composites studied in this work were processed using the raw materials listed in Table 1.

### 2.2. Experimental Methods

#### 2.2.1. Processing

The composites in this work were processed by a combination of solid-state recycling and powder metallurgy. An attempt was made to develop new metastable materials using an energy efficient method. Initially, calcium granules were ball milled in a high energy planetary ball milling machine with 10:1 ball to calcium granule mass ratio at 200 rpm, where the granules were subjected to 6 cycles of milling with an hour break between each cycle, amounting to a total processing time of 12 h. The resultant Ca powder was blended with Zn, Mn powder, and magnesium chips using the planetary ball mill, without the steel balls, for 0.5 h at 200 rpm for homogeneous mixing. The handling of the powders was done in argon atmosphere inside a glove box. This mixture was then poured into a die for cold compaction. Using a hydraulic press, the mixture was compacted for about 1 min under 1000 psi pressure. This procedure was repeated until 4 billets were retrieved from the cold compaction stage. They were then secondary processed i.e. soaked and hot-extruded, or directly hot extruded to produce rods of 8 mm diameter. These secondary processing conditions are each listed in Table 2. The composite samples are henceforth, referred to as B150, B150S, B200, and B200S, each nomenclated based on their processing conditions. Since this method used chips and powders and did not involve melting of the raw materials, it is a solid-state processing technique. This route can also be used with recycled chips and can serve as a solid-state recycling technique.

#### 2.2.2. Microstructural Characterization

The grain size analysis of the samples was performed using a Leica optical microscope model DM2500 M (Leica Microsystems (SEA) Pte Ltd., Singapore). An average of 150 grains were used to compute the average grain size of each sample. To reveal the grains, the samples were etched using the etchant: 20 mL acetic acid, 60 mL ethylene glycol, 1 mL nitric acid, and 20 mL distilled water. The secondary phases in the alloy were identified using Shimadzu LAB-XRD-6000 (Shimadzu Corporation, Kyoto, Japan) (Cu Kα; λ = 1.54056 A˚) diffractometer. The measurements were performed at scan speed: 2°/min with a scanning range: 20° to 80°. Further, the secondary phases and fracture surfaces of the samples were also analyzed using JEOL JSM-6010 PLUS/LV Scanning Electron Microscope with EDS (JEOL USA Inc., Peabody, MA, USA).

#### 2.2.3. Thermal Characterization

To determine the phase transformation temperature and ignition temperature of the alloys, Differential Scanning Calorimetry (DSC) and Thermogravimetric Analyzer (TGA) were used, respectively. 2 × 2 × 1 mm^3^ samples were heated in DSC from a temperature of 30 °C to a temperature of 600 °C at a heating rate of 5 °C/min in Ar gas. The gas flow rate was maintained at 25 mL/min in the DSC. Samples of size 2 × 2 × 1 mm^3^ were heated in TGA in purified air (flow rate: 50 mL/min) from a temperature of 30 °C to a temperature of 800 °C at a heating rate of 10 °C/min. A graph of temperature vs. time was obtained from the test. The point at which the rapid change in temperature was observed in the graph was considered as the ignition temperature of the alloy.

#### 2.2.4. Mechanical Testing

Macro-hardness tests were performed using Rockwell type B hardness tester from Future Tech FR3. The test was performed at a load of 100 kgf and an average of 5 measurements from each sample were used to find the macrohardness of the samples.

The tensile testing was performed using MTS 810 (MTS systems corporation, Eden Prairie, MN, USA) servo hydraulic tester at a strain rate of 1.7 × 10^−4^ s^−1^. This testing was performed in accordance with the ASTM standard E8M-16a on round samples with a gage diameter and length of 5 mm and 25 mm, respectively.

To determine the compressive properties, a quasi-static compressive test was performed on cylindrical samples, with height and diameter of 8 mm, on a servo hydraulic testing system MTS 810 (MTS systems corporation, Eden Prairie, MN, USA) at a strain rate of 5 × 10^−3^ min^−1^ as per ASTM test method E9-09. Five tests were conducted on each alloy for both tensile and compressive testing to ensure consistency in the results.

## 3. Results

### 3.1. Microstructure

**Figure 1:**
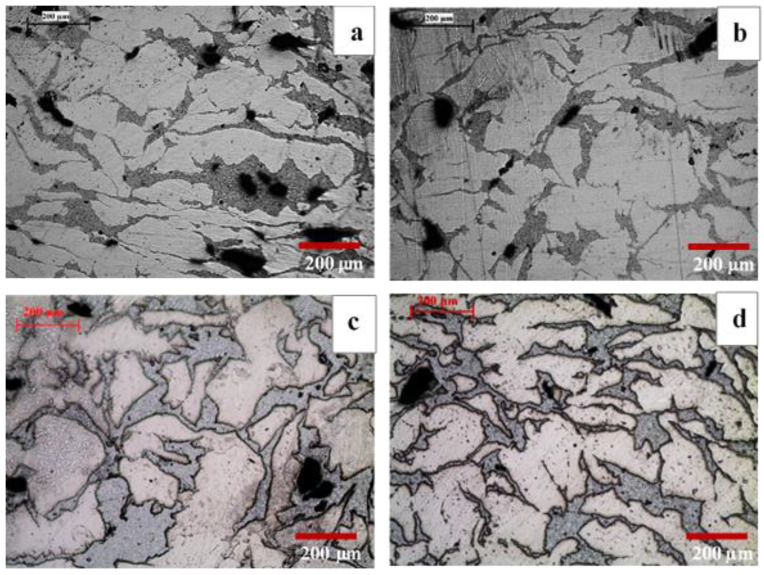
Optical micrographs of: (**a**) B150, (**b**) B150S, (**c**) B200, and (**d**) B200S samples.

**Figure 2:**
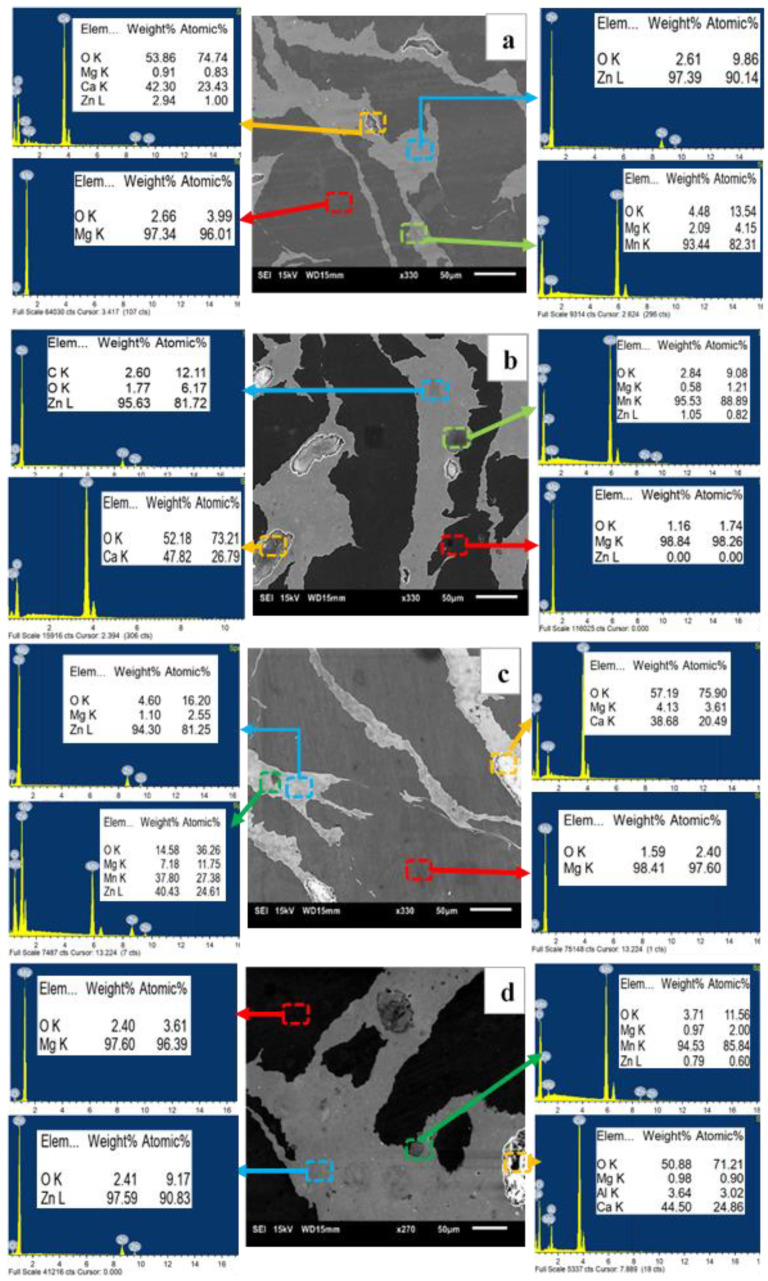
Results from EDX analysis on: (**a**) B150, (**b**) B150S, (**c**) B200, and (**d**) B200S samples.

The overall microstructure of developed composites, B150, B150S, B200, and B 200S are shown in Figure 1. From the optical micrographs, two distinctive regions, white (Mg-rich) and grey (Zn-rich) areas, can be seen in all the materials. Besides other phases in extremely limited amounts were also seen as indicated in Figure 2. The presence of pores (dark areas) are more pronounced in the materials processed at low temperature of 150 °C (Figure 1a,b) when compared to the materials processed at a higher temperature of 200 °C (Figure 1c,d). For both processing temperatures, 150 °C and 200 °C, the application of soaking on the materials does not seem to affect the pores formation.

In order to identify the formation of phases and constituent elements in white and grey areas (as seen in the optical micrographs in Figure 1) in the materials, EDX analysis was performed on the materials. The SEM micrographs superimposed with the elemental information from EDX analyses are shown in Figure 2. To note that the white and grey areas in optical micrographs correspond to the dark and bright areas in the SEM micrographs (Figure 2) respectively. Elemental information obtained from the EDX analysis showed the presence of Mg in the dark area and the presence of Zn, Ca, and Mn in the bright area in all developed materials. During processing stage, Zn, Ca, and Mn were blended and the blended powder mixture was then added into Mg chips. It is evident from the resultant microstructure (Figure 1 and Figure 2) that the blended powder mixture existed as a separate phase and reasonably well distributed within Mg phase featuring as necklace structure. To note from the EDX analysis is the appearance of high intensity oxygen peak (O) together with calcium (Ca) peaks. This indicates that Ca no longer exists in elemental condition and it appeared that there was formation of calcium oxide (CaO) in all materials. Further, both the dominant phases revealed the presence of Ca and Mn and each other indicating the diffusion of elements even at lower temperatures used in this study.

### 3.2. X-ray Diffraction Analysis

**Figure 3:**
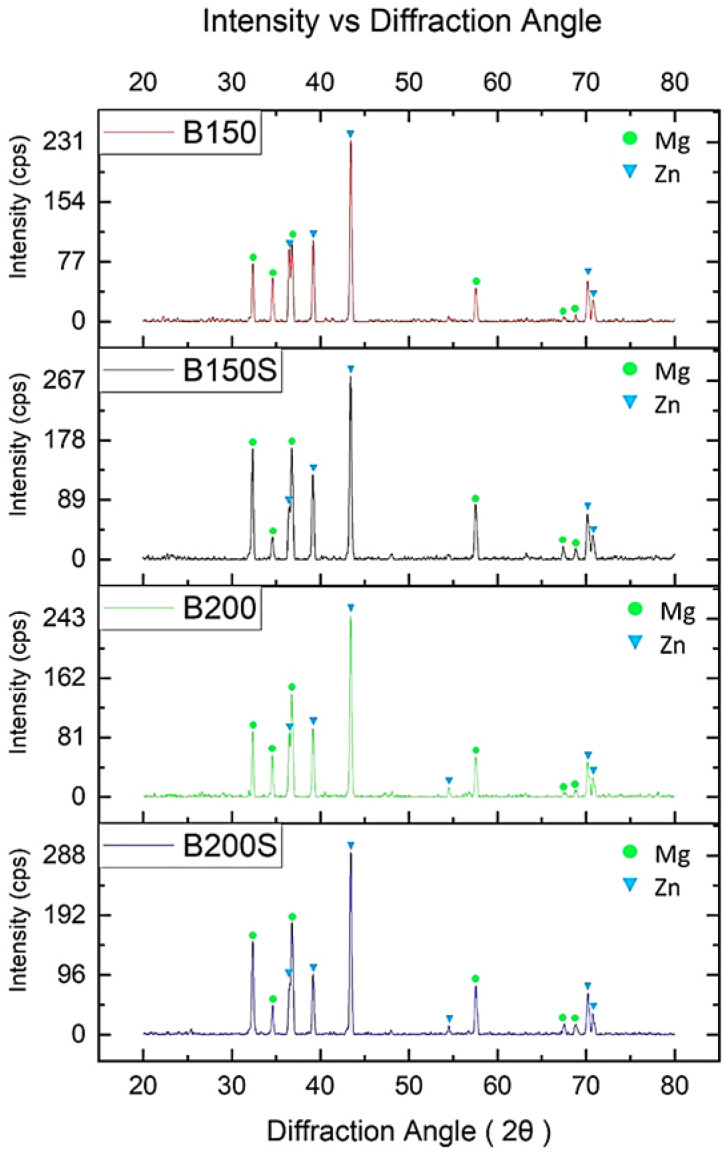
Results of XRD analysis on the developed materials.

Figure 3 shows the XRD diffractograms of the developed materials. XRD analysis revealed the peaks matching to Mg and Zn in all developed materials. The peaks related to Ca or CaO, Mn and intermetallic phases were not detected in all materials. Since low amount of Ca and Mn (1 wt.%) was added, it is reasonable that the peaks matching to those elements will not appear (vol.% < 2) in the XRD pattern of the synthesized materials. Besides, the peaks related to any other intermetallic phases were also not observed in all synthesized materials.

### 3.3. Grain Size

**Figure 4:**
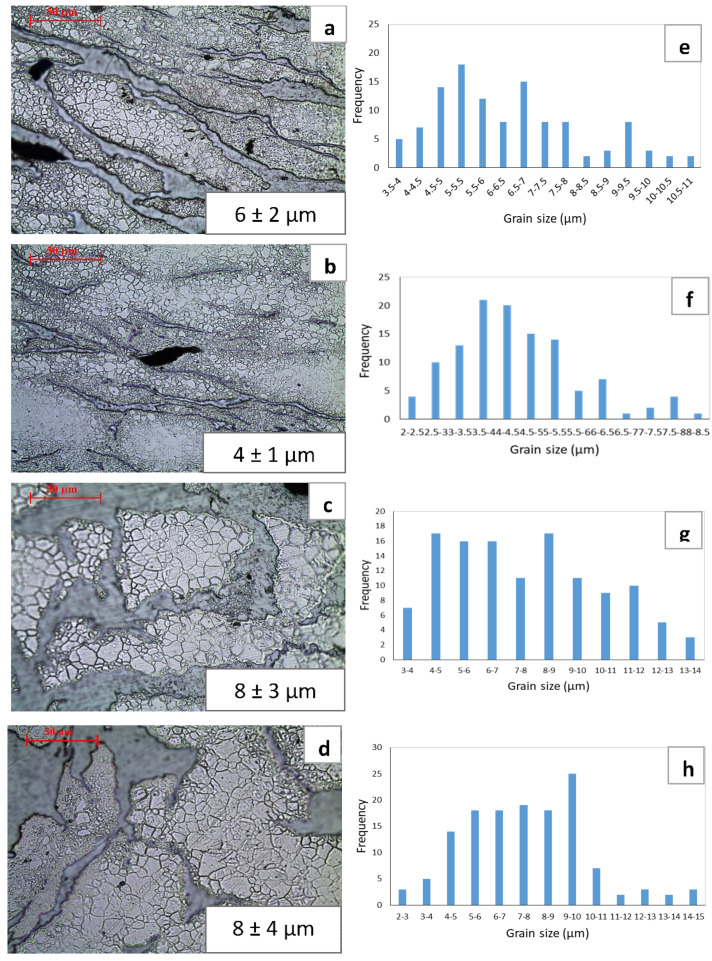
Optical micrographs showing the grain sizes in (**a**) B150, (**b**) B150S, (**c**) B200, and (**d**) B200S; and (**e**–**h**) grain size distribution charts corresponding to the micrographs presented in (**a**) to (**d**), respectively.

Figure 4 represents the grain sizes observed in the developed materials and corresponding grain size distribution histograms. Results from grain size analysis revealed the presence of finer grains in the materials processed at a lower temperature (Figure 4a,b) when compared to those processed at a higher temperature (Figure 4c,d). Between the material with soaking (heat treatment at 150 °C) and without soaking before extrusion at 150 °C, the average grain size tended to be smaller insignificantly. For the materials extruded at 200 °C, average grain size remained the same between soaked and un-soaked material. In case of materials extruded at 150 °C, heterogeneous grain size distribution was observed in both soaked and unsoaked materials (Figure 4e,f). A better and more homogeneous grain distribution was observed in the materials processed at 200 °C (Figure 4c,d,g,h).

### 3.4. Thermal Properties

**Figure 5:**
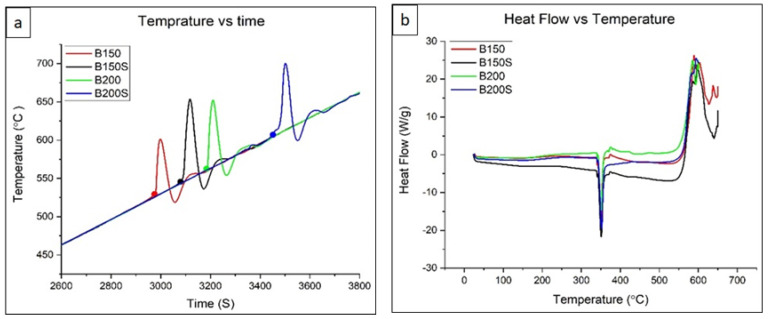
Temperature vs. Time graph from Thermogravimetric Analyzer (TGA) test indicating ignition temperature (**a**) and Heat flow vs. Temperature from Differential Scanning Calorimetry (DSC) test indicating transformation temperature (**b**).

The results from TGA and DSC analysis done on the developed materials are shown in Figure 5. The corresponding onset temperatures are tabulated and shown in Table 3. TGA results show that the ignition temperature increases with increasing extrusion temperature. The temperature also increases in the soaked materials before extrusion as compared to un-soaked materials for both heat treatment temperatures, 150 °C and 200 °C. From the DSC results, the transformation temperature in all materials is found to be the same around 338 °C. The results indicate that variation in the process parameters in terms of soaking condition and extrusion temperature has no impact on the transformation temperature of the developed materials.

### 3.5. Mechanical Properties

In order to evaluate the overall hardness of materials, the macrohardness measurement was performed on the samples. The hardness on the samples, B150, B150S, B200 and B200S, were measured to be 79 ± 1, 82 ± 1, 78 ± 1, and 75 ± 2 HRB, respectively. Taking standard deviation into consideration, similar hardness values were observed in all samples despite the different soaking conditions and extrusion temperatures. However, B150S sample showed the highest average macrohardness value.

The results from room temperature tensile tests are shown in Table 4. Overall tensile properties, 0.2% tensile yield strength (0.2% TYS), ultimate tensile strength (UTS) and ductility, decrease with an increase in processing temperature from 150 °C to 200 °C. For the materials extruded at 150 °C, a decrease in average yield and tensile strengths were observed in soaked material (B150S) when compared to unsoaked material (B150) while maintaining the same ductility. In case of the materials extruded at 200 °C, minimal difference in tensile strengths and ductility was observed regardless of soaking and unsoaking before extrusion.

The compressive response of materials is shown in Table 5. Under compressive loading, an increase in processing temperature from 150 °C to 200 °C caused a decrease in 0.2% compressive yield strength (0.2% CYS) and ductility. However, a significant increase in ultimate compressive strength (UCS) was observed in the materials processed at higher temperature, 200 °C when compared to the materials processed at lower temperature, 150 °C. Soaked and unsoaked materials showed similar compressive properties for both extrusion temperatures.

### 3.6. Fractography

**Figure 6:**
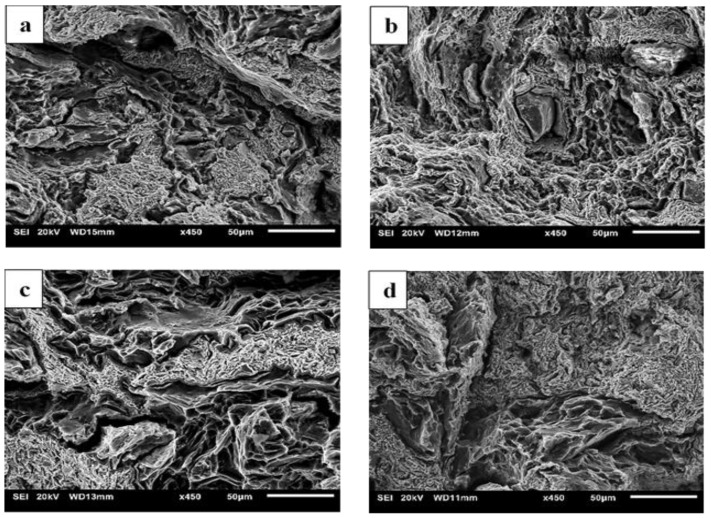
Tensile fractographs from: (**a**) B150, (**b**) B150S, (**c**) B200, and (**d**) B200S samples.

**Figure 7:**
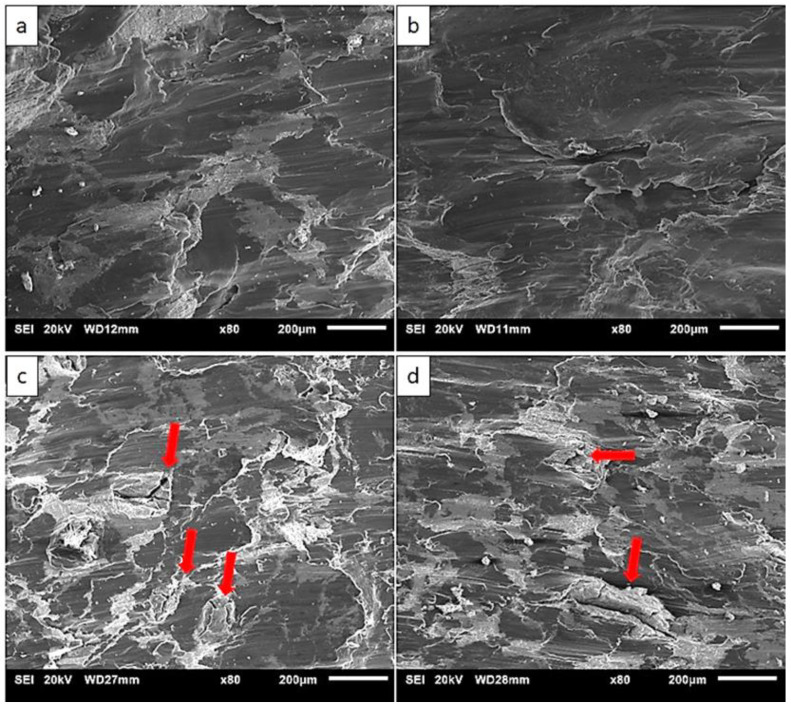
Compressive fractographs from: (**a**) B150, (**b**) B150S, (**c**) B200, and (**d**) B200S samples.

The representative fractographs taken from the fracture surfaces of failed samples under tensile and compressive loading are shown in Figure 6 and Figure 7, respectively. Regardless of the variation in soaking condition and extrusion temperature, all the developed materials showed brittle failure with numerous cracks as seen in tensile fractographs (Figure 6). From the compressive fracture surfaces, smooth fracture features with minimal cracks can be clearly seen in the materials extruded at 150 °C (Figure 7a,b). In case of materials extruded at 200 °C, cracks can be seen apparently in the fracture surfaces (Figure 7c,d). Although the soaking condition was varied, fracture features were indifferentiable between soaked and unsoaked materials under both extrusion temperatures (i.e. B150 and B150S, and B200 and B200S).

## 4. Discussion

### 4.1. Evolution of Microstructure

The microstructure of synthesized materials from the optical micrographs (Figure 1) revealed multi-phase structure. Further analyses using EDX (Figure 2) and XRD (Figure 3) confirmed that there were two dominant phases, Mg-rich and Zn-rich (containing Zn, Mg, Ca and Mn), in all samples, B150, B150S, B200, and B200S. Based on the Mg-Zn binary alloy phase diagram [20], the eutectic phase (Mg_7_Zn_3_) formation temperature is ~342 °C. The current materials were fabricated through cold compaction followed by hot extrusion at 150 °C and 200 °C with and without soaking at 150 °C and 200 °C prior to extrusion. The processing temperatures used were well below the phase formation temperature. Hence, it is coherent that the formation of intermetallic phases was not observed in the synthesized materials.

During cold compaction, cold welding among constituent metal elements—Mg chips/Mg chips, Mg chips/blended powder mixture and blended powder particles—was initiated by mechanical interlocking resulting from the deformation of metal elements under applied compaction pressure. In addition, metal–metal contact and bonding among metal elements were produced by disruption of any possible surface oxide layers due to the shear forces under uniaxial compaction. Upon application of thermomechanical processing (hot extrusion) enhanced bonding among metal elements was achieved through imposed plastic deformation. During hot extrusion, Mg chips and blended powder mixture or simply Mg and Zn could form solid state diffusion bonding (see Figure 2). Diffusion bonding between dissimilar metals forms under adequate temperature, pressure and holding time through various processing techniques including hot isostatic pressing (HIP) and high pressure torsion (HPT) [21,22,23]. The bonding temperature between dissimilar metals is suggested to be about 0.5 to 0.7 times of melting temperature [21]. In the current case, the melting temperature would be ~340 °C (Mg-Zn eutectic temperature) as the near eutectic composition of Mg and Zn was used for materials synthesis. Since the applied extrusion temperatures for current materials are 150 °C and 200 °C, the processing temperatures should be high enough to establish diffusion bonding considering the prior welding among the constituent metal elements during cold compaction, and the use of high pressure combined with shearing forces during hot extrusion [22]. Due to the involvement of holding time during processing such as in HIP and HPT, in most of the cases, intermetallic formation was unavoidable in the diffusion bonded materials. During hot extrusion of current materials, the absence of holding time negates the formation of intermetallic phases. In order to examine the phase formation in the developed composites based on the materials’ thermal profile, DSC analysis was performed as shown in Figure 5b. From the analysis, the endothermic peaks in all developed materials, B150, B150S, B200, and B200S, were found at an identical location with the same onset temperature (~338 °C). The smooth nature of the curve with no additional peak before the endothermic peak was also observed in the DSC curves. This implies that all materials showed the same and only one phase transformation temperature, ~338 °C (Table 3). Since the processing temperatures (soaking and extrusion temperature) were below ~338 °C and no phase formation was observed below 338 °C as evident from the DSC analysis, the intermetallic formation could be ruled out in the developed materials.

### 4.2. Grain Size and Distribution

It is evident that there was the effect of extrusion temperature on the grain size as can be seen in the micrographs together with grain size measurement results shown in Figure 4. Regardless of the soaking conditions, grain coarsening occurred with an average grain size of 8 µm in the materials processed at higher temperature, 200 °C when compared to those processed at lower temperature, 150 °C. For the materials processed at 150 °C, fine grains are present at Mg-Zn interface region while coarse grains were observed inside Mg matrix (Figure 4a,b). This indicates that dynamic recrystallization (DRX) took place during hot extrusion. However, it appeared that incomplete recrystallization occurred in the materials processed at low extrusion temperature (150 °C) leading to the mixture of fine and coarse grains in the microstructure (Figure 4 a,b) and the heterogeneous grain distribution as evidenced by the distribution charts (Figure 4e,l,f). Upon application of direct extrusion at 150 °C on the cold compacted material without soaking (B150), dynamic recrystallization occurred at Mg-Zn interface while a few un-recrystallized grains remained as coarse grains [24,25,26,27]. Recrystallization primarily initiated at various interfaces such as grain boundary [28,29] and interface between dissimilar metals [21,27]. In currently extruded materials, recrystallization could initiate at Mg-Zn interface and Mg grain boundaries during hot extrusion considering that the onset of recrystallization temperature of pure Mg is same as the extrusion temperature, i.e. 150 °C. However, the observation of remaining coarse grains inside Mg matrix indicates the occurrence of partial recrystallization. With soaking of the material at 150 °C for an hour before extrusion in B150S sample, similar phenomena seem to happen as the same pattern of fine and coarse grain combination (Figure 4a,b) and the same heterogeneous grain distribution (Figure 4e,f) was observed. In a study on grain crystallization of pure magnesium [29], partial recrystallization occurred at homogenization (soaking) temperature of 150 °C. Similarly, it is possible that recrystallization initiated partially even before extrusion upon application of soaking at 150° in B150S sample. As a result, fine grains were abundantly present in soaked material as seen in the distribution chart (Figure 4f) and smaller average grain size was observed in soaked material (B150S) when compared to un-soaked material (B150).

With the use of high extrusion temperature (200 °C), completely recrystallized grains can be seen in the micrographs (Figure 4c,d). This is supported by the homogeneous grain distribution pattern shown in Figure 4g,h. In fact, not only nucleation but also grain growth occurred in the materials extruded at higher temperature, 200 °C. Although the same average grain size was observed in un-soaked (B200) and soaked (B200S) material, the presence of more coarse grains was found in soaked material (Figure 4g) when compared to un-soaked material (Figure 4h). This grain coarsening could be due to the application of soaking before extrusion.

Using extrusion temperature of either 150 °C or 200 °C, soaking or not-soaking at respective temperatures has a minimal effect on the grain size variation in the extruded materials. The grain size variation is mostly dependent on the use of extrusion temperature. It also suggests that the extrusion temperature should be well above the recrystallization temperature (150 °C in this case) to get completely recrystallized grains through dynamic recrystallization process during hot extrusion.

### 4.3. Effect of Soaking and Extrusion on Igniton

From the ignition results it can be concluded that both higher soaking temperature and higher extrusion temperature had a positive impact on the ignition temperature. Overall, sample B200S demonstrated superior ignition temperature over other composites developed in this study. When extrusion die temperature is increased from 150 °C to 200 °C, the ignition temperature was seen to increase from 530 °C to 560 °C in B150 and B200 alloys, i.e., a ~ 6% increase with increase in extrusion temperature. Similarly, increase in ignition temperature from 560 °C to 606 °C i.e. a ~ 8% increase was observed by just soaking prior to extrusion. This behavior can be understood by assessing the chemistry and microstructure of the materials. In the current metal–(metal + ceramic) composites, a high amount of zinc was used (49 wt.%). Zinc is considered as an ‘ignition depressing’ alloying element to magnesium [30]. Previously, it was identified by Fassell et al. [31] that the ignition temperature of Mg-49% Zn (wt.%) alloy system reduced drastically as compared to pure Mg. Hence, the lower ignition temperature of the alloys as compared to pure Mg is attributed to the presence of Zinc. While Ca is known to improve the ignition temperature of magnesium alloys [32], since the chemical composition of the alloys is essentially same across all samples, the variation in the ignition temperature is primarily attributed to their microstructural features. With the composites demonstrating no secondary phases, the ignition behavior is attributed to the grain size and grain size distribution of the alloys. In B150 sample, fine grains were mostly observed in the optical micrograph at the interfaces of magnesium and zinc matrices (Figure 4a), resulting in heterogeneous grain distribution (Figure 4e). These fine grains reduced with increasing extrusion temperature, and B200S displayed a rather uniform grain size with relatively coarser grains (Figure 4d,h). These variations in grain size impact the oxide layer formation significantly. Since the ignition resistance of the material is determined based on the stability of the oxide layer formed which in turn depend on the grain sizes, the grain size variation is said to affect the ignition temperature. The bimodal grain boundary structure in B150 leads to a high differential in the driving force for oxidation at fine grain regions of the material. Consequently, these inhomogeneous grains lead to a non-compact and unstable oxide film, and hence are ineffective in protecting against the onset of ignition [33]. With an increase in extrusion temperature, the heterogeneous grain distribution is subsequently reduced (Figure 4). Hence, the presence of relatively uniform grains and minimal fine grains in the interface of Mg and Zn matrices facilitated the formation of a more protective oxide layer, resulting in the delay of the onset of ignition in the B200S alloy.

### 4.4. Mechanical Responses of Synthesized Materials

From the macrohardness results, there is a minimal difference in hardness among the synthesized materials. With the use of the same chemical composition in all materials and the observation of materials’ microstructures showing the same distribution pattern between Mg and blended mixture (Zn-Ca-Mn) (Figure 1), it is reasonable that all synthesized samples revealed similar level of bulk hardness. The small variation in macrohardness can be interpreted in terms of grain size variation in the materials. Especially, the highest hardness of 82 HRB was attained in the B150S sample. Among all synthesized materials, B150S exhibited the finest grains in its microstructure (Figure 4) which made it the hardest material, owing to the presence of higher grain boundary area.

From the tensile test results (Table 4), higher yield and tensile strengths were attained in the materials extruded at 150 °C when compared to those extruded at 200 °C. This can be explained by the grain size difference between the two sets of materials extruded at different temperatures. Following the common trend of strength improvement due to the grain refinement through Hall-Petch relationship, materials with fine-grained microstructures (B150 and B150S) showed higher strengths than those with coarse-grained microstructures (B200 and B200S). Between the materials with and without soaking before extrusion at respective extruded temperatures, the same level of yield and tensile strengths was found by taking standard deviation into account. However, in case of B150S, belonging to the finest grain size (4 µm) as well as the presence of fine grains more frequently (Figure 4b,f) in its microstructure, it should have shown the highest yield strength. Instead, insignificant but lower average yield strength (122 MPa) was resulted in B150S when compared to B150 (131 MPa). In fact, the yield strength value in B150S was closer to that observed in B200 (120 MPa) and B200S (118 MPa) which have larger grain size of 8 µm. Berbenni et al. [34] studied the effect of grain size distribution on the yield strength by determining the heterogeneous level of grain distribution in terms of relative dispersion. Relative dispersion is defined as ∆D/D where ∆D is the grain diameter range (D_max_–D_min_) and D is the average grain diameter and it represents grain size dispersion. The results showed the decreasing trend of yield strength with increasing relative dispersion and the grain size dispersion has significant effect on the yield strength at fine grain sizes of about 2–4 µm. For the same average grain size, yield strength decreases as the grain size dispersion increases (increasing value of relative dispersion). In a related study [35], investigation was made on the impact of grain size distribution on yield strengths of AZ31 Mg alloys. Based on the previous findings [34,35,36], the decreasing tendency of yield and tensile strengths with increasing relative dispersion was observed. It was also reported that the dispersion factor has prominent effect on the materials with fine-grained microstructure. Having fine-grained microstructure with non-homogeneous grain size distribution in B150 and B150S, the relative dispersion (∆D/D) was calculated to be 1.1 and 1.4, respectively. In the current investigation, it was also found that higher grain size heterogeneity (higher relative dispersion) observed in B150S causes the yield strength reduction when compared to B150. The results also showed that insignificant difference in relative dispersion values (1.1 and 1.4) led to a small reduction (7%) in average yield strength in B150S when compared to B150. As reported in previous investigations [34,35,36,37], yield strength variation was affected not only by the average grain size but also the grain size distribution. Hence, the current results indicate that yield strength reduction can be expected due to the existence of grain size heterogeneities in materials’ microstructure. All synthesized materials showed very low tensile ductility (Table 4) which was reflected in the fractographs (Figure 6) representing the brittle failure composed of several crackings. This could be due to the insufficient bonding among constituent metal elements [28]. This suggests that the optimization of processing parameters is needed to rectify the early failure of the samples under tensile loading.

**Figure 8:**
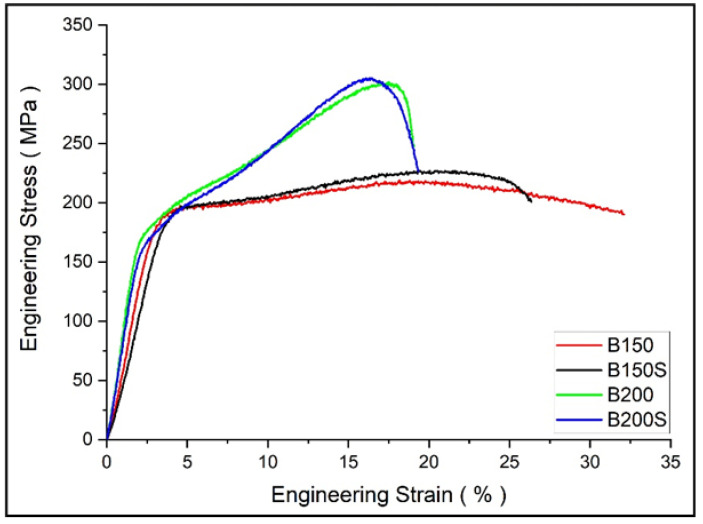
Compressive stress–strain curves of developed materials.

Compressive test results from Table 5 show higher compressive yield strength in the materials extruded at 150 °C (B150 and B150S) when compared to those extruded at 200 °C (B200 and B200S). The materials’ yield strengths are improved due to the occurrence of fine grains in B150 and B150S. It follows the general mechanism of yielding (plastic deformation) at high strength through grain refinement in previously reported Mg based composites [38,39,40,41]. However, compressive strengths were decreased in B150 and B150S when compared to B200 and B200S. Typically, under compressive loading, the main deformation mode in metals is twinning including Mg and Mg based alloys and composites. Accordingly, Mg based materials commonly deform by twinning, which is represented as concave nature in compressive stress–strain curve after yielding followed by a rapid strain hardening [37,38,39,40,41,42]. From the compressive stress–strain curves (Figure 8), materials extruded at 200 °C (B200 and B200S) reveal the compressive flow curves resembling a normal compressive stress–strain curves with rapid strain hardening after yielding. On the other hand, work hardening is relatively linear in the composites extruded at 150 °C. This led to a reduced compressive strength in B150 and B150S. Chi et al. [43] investigated the compressive response of Mg alloy with a microstructure composed of bimodal grain distribution. The alloy was fabricated using indirect extrusion intending to generate bimodal microstructure in order to study its effect on alloy’s mechanical properties. The alloy showed compressive stress–strain curve with somewhat linear strain hardening after yielding. They reported that the bimodal microstructure with incomplete recrystallization was accountable for this uncommon compressive response. In the current investigation, the main difference between two sets of materials extruded at 150 °C and 200 °C is the evolution of microstructure, heterogeneous grain distribution in the former and homogeneous grain distribution in the latter case. Consequently, the microstructural heterogeneity of the materials extruded at 150 °C led to inhomogeneous strain distribution during plastic deformation under compressive loading. For Mg based materials with weak texture, the basal planes were not sufficiently aligned to cause the tensile twinning during compressive deformation. Whereas, for those with strong texture, twin initiation and propagation was easily accommodated by the preferred basal orientation. Meng et al. [29] reported the presence of weak texture in fine, DRXed grains at the initial stage of recrystallization. Based on the previous studies [29,43], in B150 and B150S, only a few coarse grains accommodated twinning deformation while twinning was obstructed in fine and weakly textured grains during plastic deformation after yielding. During the strain hardening stage, plastic flow could continue with slip instead of twinning deformation in fine DRXed grains. Accordingly, post-yielding deformation seems to be accommodated by insufficient twinning together with slip causing the linear strain hardening in materials extruded at 150 °C [43]. Regarding compressive ductility, materials extruded at 200 °C exhibited lower ductility when compared to those extruded at 150 °C. In case of materials extruded at 200 °C, the main deformation mode is twinning and failure follows upon exhaustion of twin activities. Besides, from the fractographs (Figure 7c,d), the formation of cracks were found inside blended mixture area (Zn-Ca-Mn) yet no cracking was observed at Mg sites. It appears that there is flow incompatibility between Mg and blended mixture during compressive deformation which could also be the cause for a reduced ductility in B200 and B200S. In case of materials extruded at 150 °C, the deformation seems to be dominated mainly by slip. To note that the shape of the compressive stress–strain curves matches with the tensile stress–strain curves in this respect. In addition, fractographs (Figure 7a,b) showed minimal cracking with smooth flow conforming the occurrence of higher ductility.

## 5. Conclusions

The summary of findings from the current investigation is listed as follows:

1. No evidence of intermetallic formation was observed in all developed metal–(metal + ceramic) composites fulfilling the aim of current investigation. This made the currently established sinter-less PM method to be suitable for synthesis of metastable metal–(metal + ceramic) composites using either Mg or Zn as matrix material.
2. With the use of low extrusion temperature (150 °C), fine recrystallized grains were abundantly found in the composites. Whereas coarsening of recrystallized grains was observed at high extrusion temperature (200 °C). Due to negligible difference in grain size between soaked and un-soaked materials it can be inferred that soaking condition (with or without soaking prior to extrusion) has minimal effect on the grain size variation and it is mainly influenced by extrusion temperature.
3. Based on the grain distribution analysis, heterogeneous grain distribution was observed in the composites processed at low temperature (B150 and B150S) whereas a relatively more homogeneous grain distribution was observed in those processed at high temperature (B200 and B200S).
4. Thermal analysis using TGA revealed the highest ignition temperature in B200S. The presence of relatively uniform grains in B200S facilitated the formation of a more protective oxide layer delaying the onset of ignition. Large grain size variations observed in B150 and B150S caused an adverse effect on ignition temperature.
5. The bulk hardness assessed in terms of Rockwell hardness test revealed similar level of macrohardness. A small variation in average hardness follows the grain size variation while the highest hardness of 82 HRB was realized in B150S which displayed finest average grain size.
6. Between B150 and B150S, despite having finer grains, B150S showed insignificant but a decrease in average tensile yield strength. A higher level of grain size heterogeneity in B150S is accountable for the yield strength reduction. Regardless of the extrusion temperature or soaking condition, all synthesized composites showed brittle nature with low tensile ductility of 2.4–3.4% under tensile loading.
7. The existence of finer grains provided compressive yield strength increment in B150 and B150S when compared to B200 and B200S. However, post yielding deformation was influenced by the grains heterogeneity causing relatively linear strain hardening with a reduced compressive strength in B150 and B150S. Whereas common phenomenon of rapid strain hardening during plastic deformation with an increase compressive strength was realized in B200 and B200S.

## Figures and Tables

**Table 1:** List of raw materials.

Materials	Supplier	Purity (%)	Form
Magnesium	Acros Organics (Fair Lawn, NJ, USA)	>99.9	Chips
Zinc	Good Fellow (Huntingdon, UK)	98.8	Powder
Manganese	Good Fellow (Huntingdon, UK)	>97.5	Powder
Calcium	Merck (Singapore)	99	Granules

**Table 2:** Secondary processing conditions of the compacted Mg_49_Zn_49_Mn_1_Ca_1_ billets.

Sample	Soaking Temperature (°C)	Soaking Time (h)	Extrusion Temperature (°C)
B150	-	-	150
B150S	150	1	150
B200	-	-	200
B200S	200	1	200

**Table 3:** Measured results from TGA and DSC.

Material	Ignition Temperature (°C)	Phase Transformation Temperature (°C)
B150	530	338.5
B150S	545	338.5
B200	560	338.9
B200S	606	338.8

**Table 4:** Room temperature tensile test results.

Material	0.2% TYS (MPa)	UTS (MPa)	Ductility (%)
B150	131 ± 18	178 ± 3	3.4 ± 1.5
B150S	122 ± 3	172 ± 17	3.4 ± 1.8
B200	120 ± 2	142 ± 8	2.4 ± 0.3
B200S	118 ± 13	143 ± 13	2.5 ± 1

**Table 5:** Room temperature compression test results.

Material	0.2% CYS (MPa)	UCS (MPa)	Ductility (%)
B150	186 ± 3	219 ± 1	33 ± 5
B150S	185 ± 6	217 ± 9	26 ± 2
B200	175 ± 3	308 ± 1	21 ± 3
B200S	169 ± 9	314 ± 8	20 ± 2
